# Proteome profiling of gestational diabetes mellitus at 16‐18 weeks revealed by LC‐MS/MS

**DOI:** 10.1002/jcla.23424

**Published:** 2020-06-15

**Authors:** Xiaoting Liu, Jingru Sun, Xinyu Wen, Jinyan Duan, Dandan Xue, Yuling Pan, Jinghua Sun, Wei Zhang, Xiaoliang Cheng, Chengbin Wang

**Affiliations:** ^1^ Medical School of Chinese PLA & Medical Laboratory Center First Medical Center of Chinese PLA General Hospital Beijing China; ^2^ Qlife Lab Co., Ltd Shenzhen China

**Keywords:** co‐regulated area, gestational diabetes mellitus, global correlation, predictive biomarker, proteome

## Abstract

**Background:**

The practices used to diagnose gestational diabetes mellitus (GDM) could only be carried out around the time of detectable symptoms, and predictive capacity is little.

**Methods:**

LC‐MS/MS was conducted to explore overview proteomics for GDM complicated pregnant woman at 16‐18 gestation weeks, while normal pregnant for control. Enzyme‐linked immunosorbent assay was further applied in an independent cohort of 15 GDM cases and 15 controls for verification.

**Results:**

The results indicated that 24 protein expression levels were significantly changed in GDM group samples, and inflammation, oxidative stress, insulin resistance, blood coagulation, and lipid homeostasis were associated with GDM. The abnormal expression of CRP and IGFBP2 was verified in the first‐trimester maternal plasma in women who subsequently developed GDM.

**Conclusions:**

This study not only identified 24 potential predictive biomarkers for GDM also provided a global overview of protein rearrangements induced by GDM.

## 1 INTRODUCTION

Gestational diabetes mellitus (GDM) is defined as “any degree of glucose intolerance with onset or first recognition during pregnancy”.^1^ GDM is a common complication of pregnancy, and the prevalence is continuously increased, especially in Asia.^2^ GDM is clinically important because of its devastating impacts on the developing child and the expectant mother. For pregnant woman, GDM would cause maternal complications, increase adverse perinatal outcomes,^3^ and add risk for developing type‐2 diabetes in later life.^4^ Prenatal exposure to gestational diabetes increases risks of macrosomia, neonatal hypoglycemia, hyperbilirubinemia, shoulder dystocia, birth trauma, metabolic syndrome, and obesity in later life for child.^5^, ^6^, ^7^

GDM screening and diagnosing practice include one‐ and two‐step approaches. Two‐step approach contains 50 g oral glucose challenge test and 100 g 3‐hour oral glucose tolerance test (OGTT), and one‐step approach uses a 75 g 2‐hour OGTT.^8^, ^9^ For 50 g oral glucose challenge test, two thresholds are used (130 and 140 mg/dL).^8^ The problem is that the practices used to diagnose GDM could only be carried out around the time of detectable symptoms and predictive capacity is little. At 24‐28 gestation weeks, when GDM diagnosis carried out, harms may have potentially occurred to both mother and child.^10^ Effective intervention and management could positively affect maternal and fetal outcomes; therefore, predictive biomarker exploration is desirable.

Previous studies investigated the potential value of first‐trimester maternal serum markers of GDM reported promising results, such as insulin resistance (sex hormone‐binding globulin and homeostasis model assessment index), inflammation (high sensitive C‐reactive protein), and adipocytokines (adiponectin, visfatin, and leptin) which have been measured in the first or second trimester of GDM.^11^, ^12^, ^13^, ^14^, ^15^

Mass spectrometry‐based proteomics of human plasma could provide a more profound understanding in the pathogenesis of many diseases, such as hypertension,^16^ cancer,^17^ GDM,^18^ and major depressive disorder.^19^ The above studies were based on that proteins secreted into human body fluid could reflect disease states, and alterations in expression level are indicative of developing lesion. However, due to the complexity and high dynamic variation of the human plasma components, the overview proteomics of GDM remain challenging.

The aim of this study was to explore the proteomics of GDM for further understanding the subsequent development of gestational diabetes. In our study, protein expression levels and global correlation analysis including inflammation, oxidative stress, insulin resistance, blood coagulation, and lipid homeostasis were investigated in GDM and normal pregnancy by liquid chromatography‐tandem mass spectrometry (LC‐MS/MS) analysis.

## 2 MATERIAL AND METHODS

### 2.1 Patient demography

Clinical characteristics of both the GDM and control samples are shown in Table 1. The mothers with GDM were slightly older and had higher pre‐pregnancy BMI values when compared with the control group. There was no significant difference in gestational weeks between the two groups. OGTT of fast, 1, and 2 hours was all significantly different.

**Table 1:** Clinical characteristics of control and GDM women

Variable	Control	GDM	*P*‐value
Sample size	22	22	/
Maternal age (y)	29.23 ± 2.13	30.73 ± 2.56	.045
Pre‐pregnancy BMI (kg/m^2^)	20.78 ± 1.17	24.14 ± 4.70	.003
Gestational weeks	38.95 ± 1.19	38.41 ± 1.70	.234
OGTT, fast (nmol/L)	4.17 ± 0.20	5.03 ± 0.63	1.12E‐05
OGTT, 1 h (nmol/L)	6.59 ± 1.28	10.32 ± 1.45	2.5807E‐09
OGTT, 2 h (nmol/L)	6.08 ± 0.93	8.43 ± 1.79	4.611E‐05

Note

Date presented as mean ± SD.

### 2.2 Chemical and reagents

Urea, dithiothreitol (DTT), iodoacetamide (IAA), ammonium bicarbonate, trifluoroacetic acid (TFA), and SOLA HRP 96‐well SPE plate were bought from Thermo Fisher (Thermo Fisher Scientific). Trypsin was bought from Promega (Promega). Lysyl endopeptidase (Lys‐C) was bought from Wako (Wako). All organic reagents used in the experiment are chromatographically pure reagents. The experimental water is Milli‐Q ultrapure water (Millipore). Other reagents are analytically pure reagents unless otherwise indicated.

### 2.3 Sample collection and storage

A total of 44 blood samples were involved in this study, including 22 GDM patients and 22 controls at 16‐18 weeks. Clinical data on the outcome of pregnancy were collected until delivery for the 44 subjects. All GDM patients were diagnosed with oral glucose tolerance test (OGTT) during 24‐28 weeks of gestation in Chinese PLA General Hospital. Subjects were considered to have GDM according to two criteria: first, fasting morning venous plasma glucose reached or exceeded 5.1 mmol/L; second, venous plasma glucose levels reached or exceeded two or more of the following values: a fasting morning plasma glucose of 5.1 mmol/L; a 1 hour post‐load glucose of 10.0 mmol/L; a 2 hour post‐load glucose of 8.5 mmol/L. Healthy controls were selected based on similar gestational weeks and gestational age to maintain similar maternal baseline characteristics. Subjects who displayed a history of type‐2 diabetes were excluded, as were smokers or those with a chemical dependency, and those who exhibited fetal congenital anomalies or any other confounding pathology (including hyperthyroidism, hypothyroidism, hypertension, and hyperlipidemia). The approval of this study was granted by Chinese PLA General Hospital Ethics Committee and met the Declaration of Helsinki.

### 2.4 Plasma protein digestion

For each sample, 1 μL (about 60 μg proteins) plasma was added to 30 μL 8 mol/L urea, (with 50 mmol/L ammonium bicarbonate) for denaturation in a 96‐well plate format. Dithiothreitol (DTT) was added to final concentration of 10 mmol/L and incubated at room temperature for 30 minutes. Iodoacetamide (IAA) was added to final concentration of 20 mmol/L and incubated in darkness at room temperature for 30 minutes. Then, DTT was added to final concentration of 10 mmol/L to terminate alkylation. After denaturation and alkylation, proteins were digested with Lys‐C (Wako) in an enzyme/protein ratio of 1:100 (w/w) for 2 hours. 210 μL of 50 mmol/L ammonium bicarbonate was added to dilute 8 mol/L urea to 1 mol/L urea and digest with trypsin (Promega) in an enzyme/protein ratio of 1:50 (w/w) overnight. Peptide digest was desalted with SOLA HRP 96‐well SPE plate (Thermo Fisher Scientific). In brief, peptides were first loaded onto SOLA HRP and then washed with 0.1% trifluoroacetic acid (TFA) and finally eluted with 30% acetonitrile (ACN) and 60% ACN containing 0.1% TFA. Elute was lyophilized in a vacuum centrifuge for LC‐MS/MS proteome analysis.

### 2.5 LC‐MS/MS analysis

Plasma peptides were dissolved in loading buffer (containing 0.1% formic acid). Peptides (~2 µg) were loaded onto a trap column of Acclaim PepMap RSLC C18 column, 2 μm, 100 Å, 75 μm i.d. × 20 mm (Thermo Fisher Scientific) and separated with an analytical column of Acclaim PepMap RSLC C18 column, 2 μm, 100 Å, 75 μm i.d. × 25 cm (Thermo Fisher Scientific) using an EASY‐nLC 1200 system (Thermo Scientific). Buffer A (0.1% formic acid in water) and buffer B (0.1% formic acid in 80% CAN) were used to separate the peptides at a segmented gradient and flow rate. The segmented gradient was 0‐4 minutes, 1%‐8% B, 4‐80 minutes, 8%‐30% B, 80‐86 minutes 30%‐90% B, 86‐90 minutes 90%‐1% B, 90‐95 minutes 1% B, and the segmented flow rated was 0‐86 minutes, 500 nL/min and 86‐95 minutes, 600 nL/min. The column temperature was 55°C.

Data‐independent acquisition (DIA) was used on Orbitrap Fusion Lumos Tribrid MS (Thermo Scientific). Each MS cycle contained one full MS and 60 DIA scans. Cycle time was ~4.8 seconds. Sixty DIA isolation windows varied according to peptides *m/z*. The full scan was acquired with a resolution of 6000 at *m/z* 200, recording window between 350‐1200 *m/z*, automatic gain control target of 2 × 10^5^, a max injection time of 20 ms and normalized collision energy of 32%. The 60 DIA variable windows were acquired with a resolution of 30 000 at *m/z* 200, recoding window between 200 and 2000, automatic gain control target of 5 × 10^5^, a max injection time of 55 ms.

### 2.6 ELISA analysis

ELISA analysis was performed to verify the quantitative proteomics results. First‐trimester maternal plasma samples from an independent cohort of pregnant women (15 with GDM cases and 15 controls) were analyzed using commercially available kits for C‐reactive protein (CRP) (#SEKH‐0138, Solarbio Life Sciences, Corp.) and insulin‐like growth factor‐binding protein 2 (IGFBP2) (#SEKH‐0213, Solarbio Life Sciences, Corp.) according to the manufacturer's instructions.

### 2.7 Data analysis

All DIA files were extracted from home generated plasma library containing 73 466 precursors, 55 433 modified peptides and 5190 proteins using Spectronaut X (Biognosys). Raw MS data files were converted to HTRMS files with HTRMS converter (Biognosys). HTRMS files were imported to Spectronaut with default parameters with the decoy generation set to “mutated.” Cutoff of fold change > 1.3 or <0.3785 and Q < 0.05 (FDR‐corrected *P* value for *t* test) was set for differentially expressed proteins. Correlation and ROC analysis were carried out using R software version 3.5.3.

### 2.8 Bioinformatics analysis

Gene Ontology (GO) analysis and UniProt‐KB keyword analysis for the significantly changed proteins and correlated proteins were conducted on the Database for Annotation, Visualization and Integrated Discovery (DAVID) version 6.8.^20^, ^21^ Fisher's exact test was used in determining the significant enrichment terms, and .05 was set as the threshold (*P*‐values). Only significantly changed category terms were reported in this study.

Inflammation system proteins included proteins with Uniprot‐UK keywords for inflammatory response, immunity, innate immunity, complement pathway, complement alternate pathway, acute‐phase, membrane attack complex, cytolysis, and antimicrobial.

Uniprot‐UK keywords for high‐density lipoprotein (HDL), low‐density lipoprotein (LDL), very‐low‐density lipoprotein (vLDL), lipid transport, and chylomicron were used for lipid homeostasis system protein filtering. SAA1, SAA2, SAA4 were excluded.

## 3 RESULTS

### 3.1 Global profiling of plasma proteins in GDM and control samples

**Figure 1:**
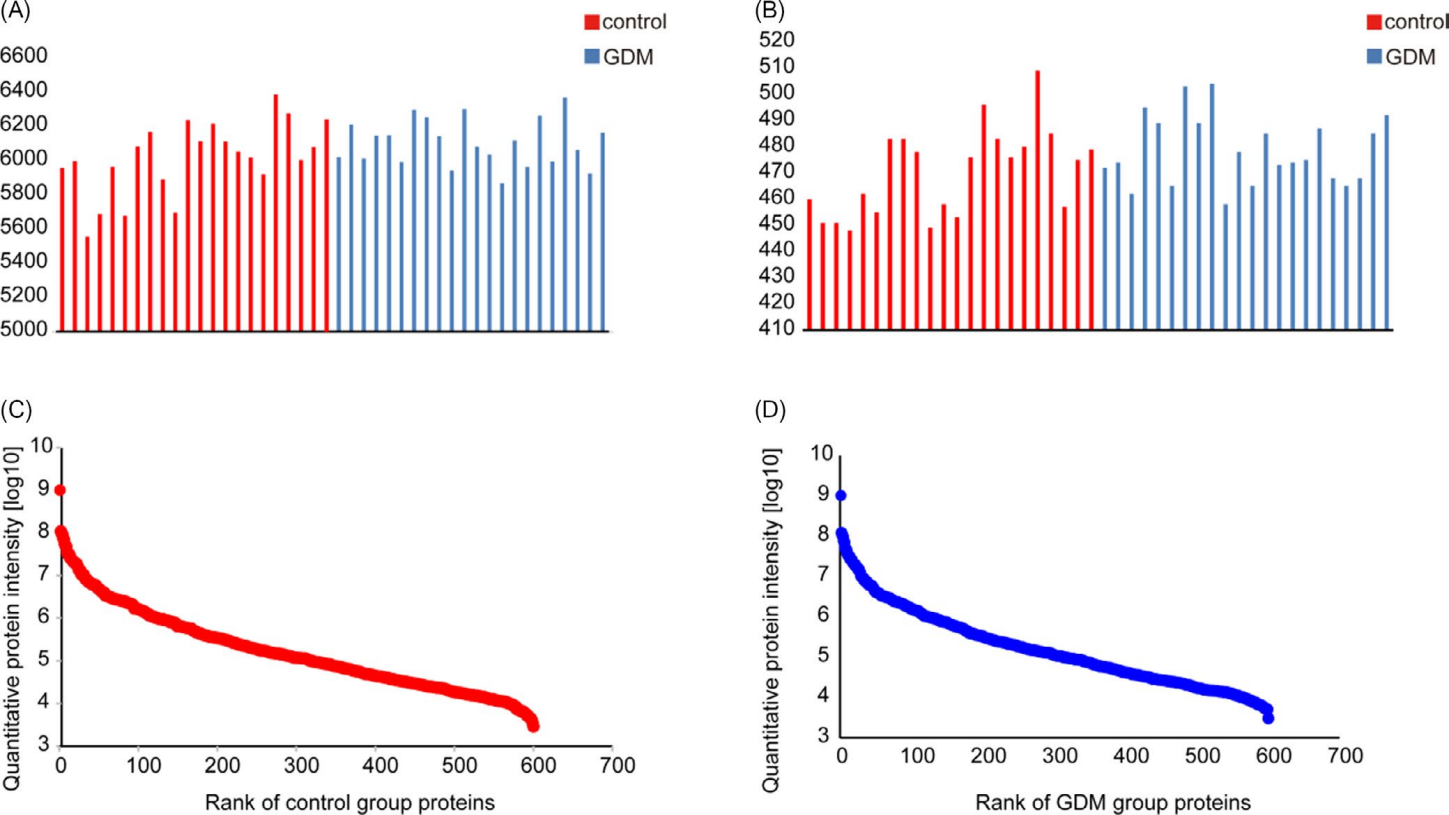
Identified and quantified peptides and proteins. A, identified and quantified peptides; B, identified and quantified proteins; C, rank of quantified proteins in control group; D, rank of quantified proteins in GDM group

To discover plasma protein alternations induced by GDM, global proteomic profiling of 22 patients and 22 healthy controls were analyzed using LC‐MS/MS with DIA data acquisition method. High‐accuracy LC‐MS/MS was used to identify and quantitatively detect a large scale of proteins. With DIA acquisition method, high abundant proteins were not depleted. Among these samples, 6058.9 peptides (5555‐6385, Figure 1A) and 474.4 protein groups (448‐509, Figure 1B) per sample were detected and quantified on average in this study. These proteins are listed in Table S1. Ranked proteins in control and GDM group indicated similar distributions in plasma proteome (Figure 1C,D).

### 3.2 GDM induced plasma proteome rearranges

**Figure 2:**
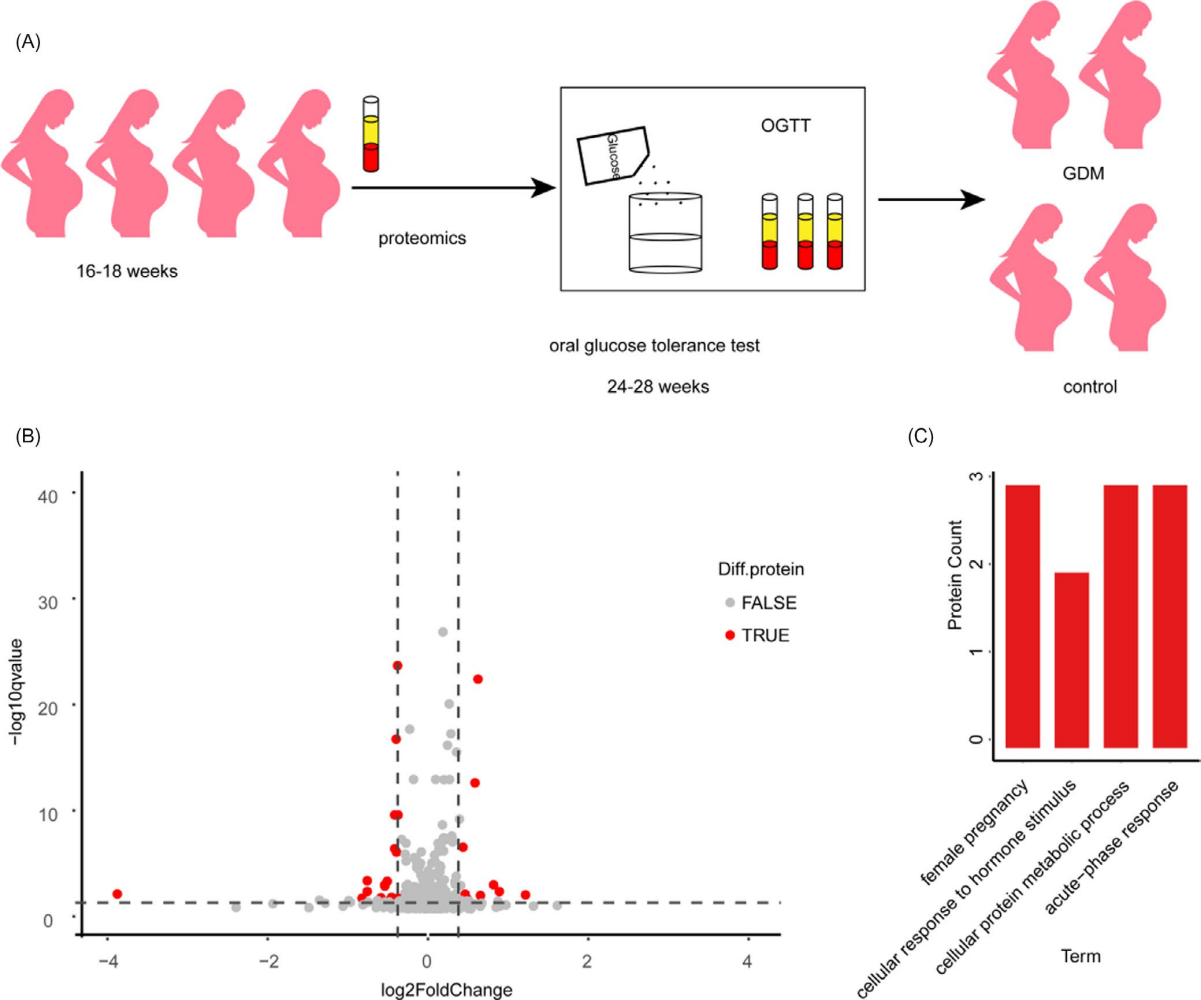
Study design and significantly changed proteins. A, study design; B, volcano plot for control and GDM group; C, significantly enriched GOBP

Our aim was to reveal potential biomarkers to predict GDM at early second‐trimester through plasma proteomic analysis. The study design is illustrated in Figure 2A. Through fold change and t test analysis, 24 proteins were found to change significantly (Figure 2B and Table S2) in GDM samples. Levels of 16 proteins decreased and 8 proteins increased in GDM samples. Increased proteins induced by GDM included alpha‐N‐acetylglucosaminidase, poliovirus receptor, C‐reactive protein, proteoglycan 4, serum amyloid p‐component, serum amyloid A‐2 protein, and growth hormone receptor fibrinogen alpha chain. Decreased proteins included three groups of pregnancy‐related proteins (pregnancy zone protein, pregnancy‐specific beta‐1‐glycoprotein 2, and insulin‐like growth factor‐binding protein 2), three groups of immunoglobulins (Ig lambda chain V‐III region LOI, Ig mu chain C region, and Ig heavy chain V‐I region V35), four enzymes (serum paraoxonase/lactonase 3, trypsin‐3, Xaa‐Pro dipeptidase, and citrate lyase subunit beta‐like protein in mitochondrial), two groups of binding proteins (sex hormone‐binding globulin and cofilin‐1), one transcription factor (zinc finger protein basonuclin‐2) and three other proteins including secreted phosphor protein 24, isthmin‐2, and programmed cell death 6‐interacting protein.

We annotated the 24 significantly changed proteins with gene ontology (GO). GOCC (cell component) indicated that these proteins located in extracellular, blood microparticle, and cell surface (Table S3). Significantly enriched GOBP (biological process) included acute‐phase response, female pregnancy, cellular proteins metabolic process, and cellular response to hormone stimulus (Figure 2C). And based on Fisher's exact test, 4 keywords were obtained (*P* ≤ .5) for GOMF (molecular function) including complement component C1q binding, virion binding, endopeptidase inhibitor activity, and protein homodimerization activity (Table S3). As the complicated relationship of these proteins, we set out to exploit global co‐regulation proteins.

### 3.3 Changed proteins have diagnostic value

**Figure 3:**
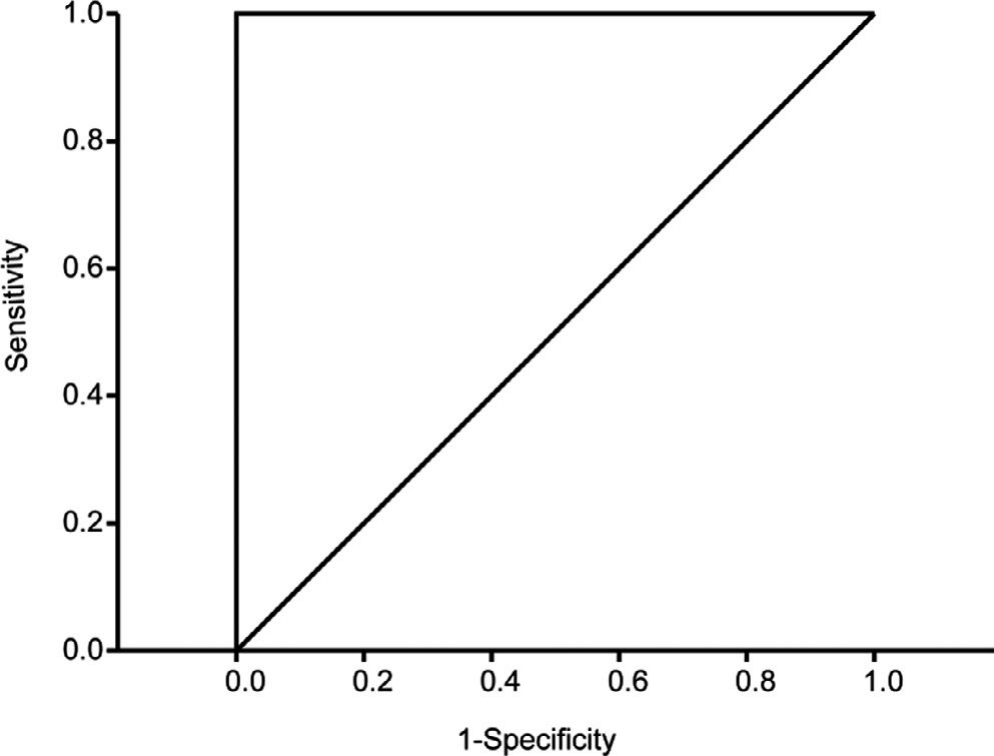
Receiver operating characteristic analysis on the changed proteins

To examine the potential diagnostic value of the changed proteins, receiver operating characteristic (ROC) analysis was performed. ROC curve was constructed between OGTT diagnose results of subjects and binary logistic regression predictions on the two‐category attribute based on the 24 changed proteins. The area under the curve (AUC) was 1 (Figure 3) and indicated these proteins as a perfect classifier.

### 3.4 Global correlation maps reveal protein co‐regulations

**Figure 4:**
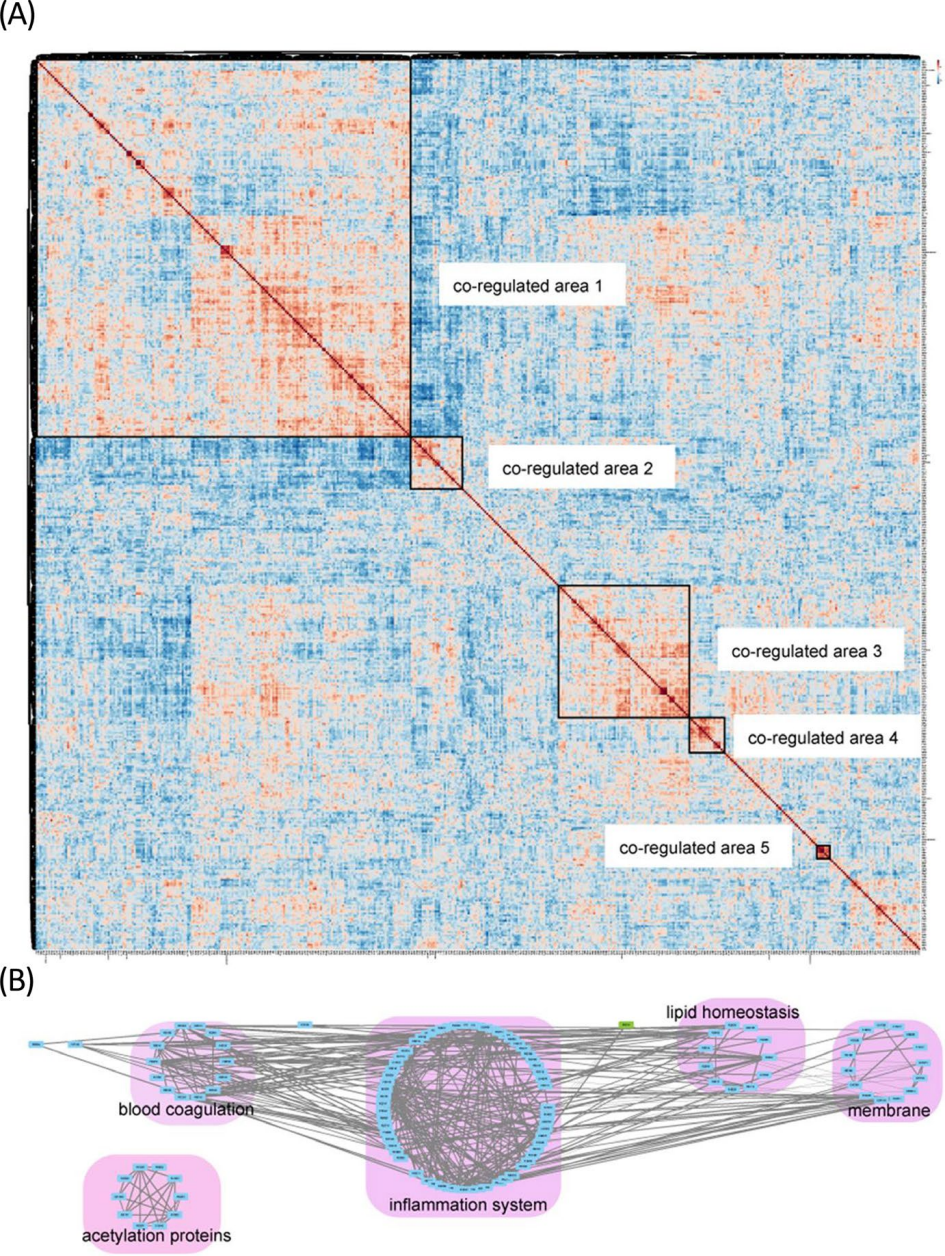
Global correlation map of the plasma proteome. A, global correlation map; B, correlation network analysis of the main physiological process proteins in clusters in A

Proteins work together in a complex network in biosystems, and the concentrations and activities of proteins in the network are rigorously controlled. Therefore, after comparison of GDM and control samples, we investigated the co‐regulated proteins to explore pathological mechanism of GDM. At least 50% proteins that quantified in samples were included. Pairwise correlations between these proteins and four clinical parameters were calculated to construct global correlation heatmap (Figure 4A). In the correlation heatmap, proteins and clinical parameters were grouped with hierarchical cluster analysis and correlation coefficients were coded as colors.

The global correlation map was constructed with 475 proteins, faster OGTT, 1 hour OGTT, 2 hours OGTT, and BMI values. Protein abundant levels, OGTT results, and BMI generated a matrix of 114 481 correlation coefficients (Figure 4A and Table S4). As previous studies indicated that proteins and clinical parameters associated with the same underlying regulatory mechanism would cluster in the same area, we used this map to explore GDM induced co‐regulated proteins and clinical parameters.

Biologically related proteins were found to co‐regulate in the correlation map. Strongly co‐regulated proteins and clinical parameters formed co‐regulated areas were all small in this study. When we pay attention to the relationship of proteins in and out of a co‐regulated area and HCA clustering, generally five protein co‐regulated areas were observed. Uniprot‐UK keyword annotation was used to interpret the functions of these proteins. The largest co‐regulated area was as large as 200 × 200, including faster, 1, 2 hours OGTT, BMI values, and 197 proteins (Figure 4A). This largest module contained 52 inflammation system proteins, 18 lipid homeostasis system proteins, 19 blood coagulation proteins, 6 apolipoproteins, 4 antioxidative stress proteins (extracellular superoxide dismutase, peroxiredoxin‐1, serum haptoglobin, and paraoxonase/arylesterase 1), and 1 insulin resistance‐related protein (C‐reactive protein). Co‐regulated area 2, which was negatively correlated with co‐regulated area 1, contained 10 immunoglobulin, 8 membrane proteins, and some other proteins. Co‐regulated area 3 showed different relationship with proteins and clinical parameters in co‐regulated area 1. Co‐regulated area 3 included 26 inflammation system proteins, 17 membrane proteins, 4 blood coagulation proteins, and some other proteins. Co‐regulated area 4 included 12 acetylation related proteins and 7 other proteins. Co‐regulated area 5 included four hemoglobin subunits, 2 peroxidase, and 1 carbonic anhydrase.

Correlation network of the Uniprot‐UK keyword annotated proteins in the five co‐regulated areas revealed the connections between these proteins. Only proteins with absolute correlation coefficients values above 0.5 were selected to display their relationships (Figure 4B and Table S5). This network highlighted the connections of the main physiological processes. Strong correlations were observed within inflammation system proteins, and the correlations of inflammation with blood coagulation, lipid homeostasis, and membrane proteins were also captured. Two antioxidative stress protein (P08294, P27169) showed correlation with blood coagulation, inflammation system and lipid homeostasis. Acetylation proteins only showed correlations within these proteins and did not show correlation with the other proteins.

### 3.5 Protein expression verificated by ELISA

**Figure 5:**
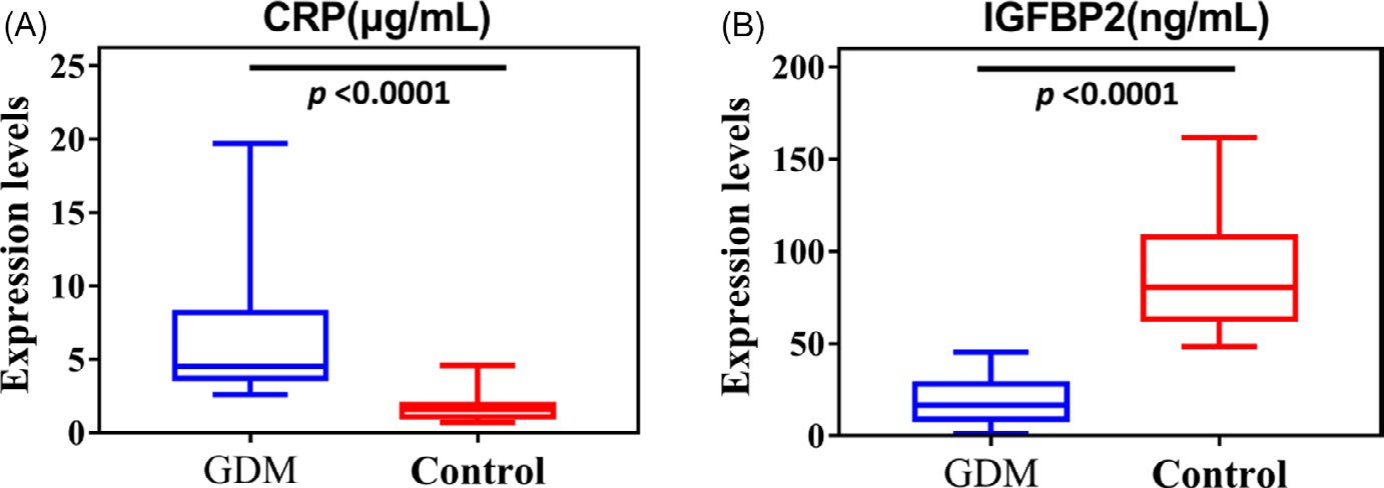
Box plot of CRP (A) and IGFBP2 (B) results of GDM and the control groups (*P*‐value < .0001)

**Figure 6:**
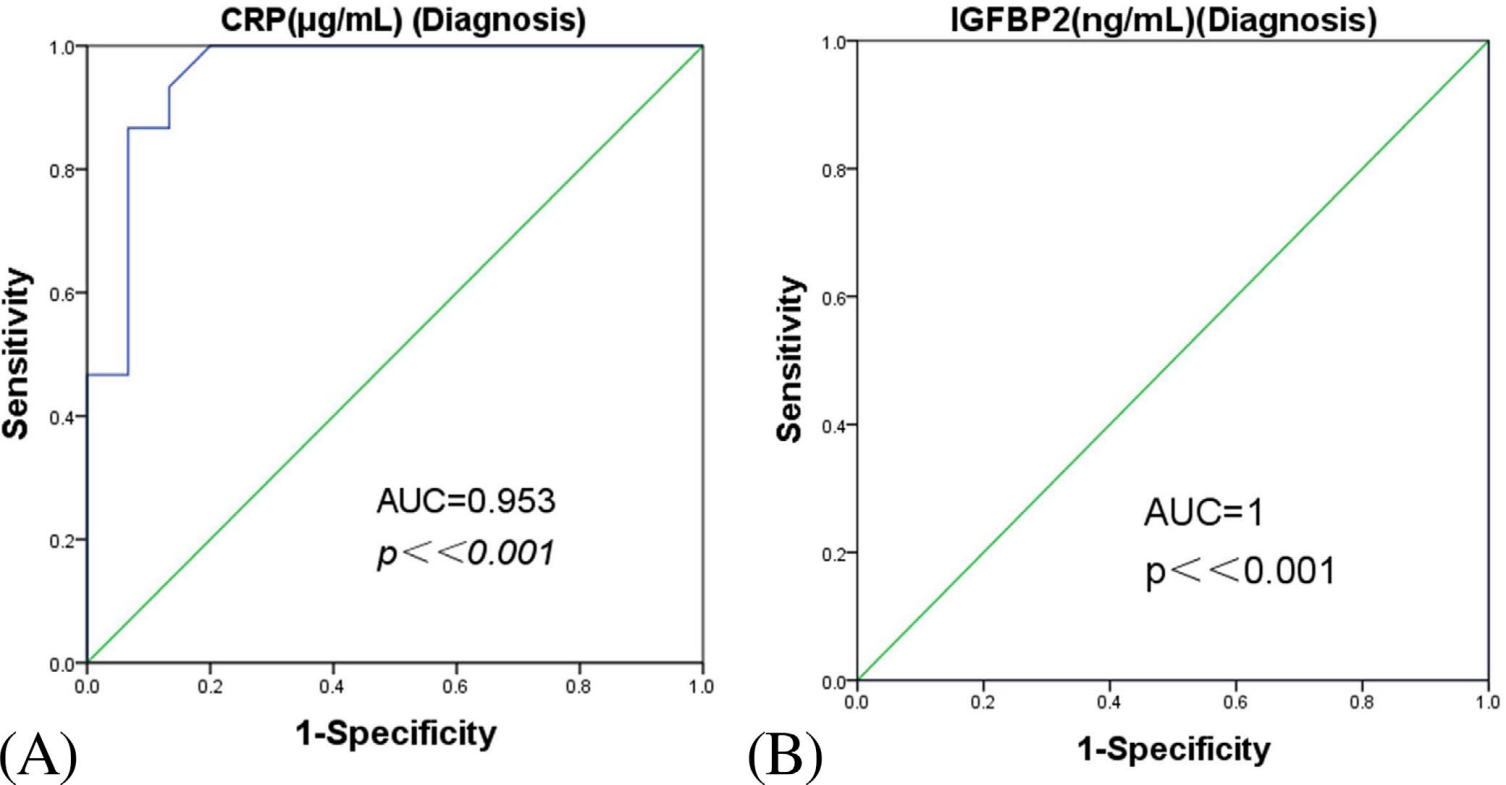
Receiver operating characteristic curves showing the performance of CRP (A) and IGFBP2 (B) as individual diagnostic biomarkers

Consistent with the LC‐MS/MS data, ELISA analysis verified the abnormal expression of CRP and IGFBP2 in the first‐trimester maternal plasma in women who subsequently developed GDM (Figure 5). Both proteins were examined for their performance in differentiating between GDM and control samples. The area under the curve (AUC) obtained for CRP was 0.953 and for IGFBP2 1.0 at *P* < .001 (Figure 6).

## 4 DISCUSSION

Gestational diabetes mellitus could alter human metabolism, and the potential molecular mechanisms of GDM remains unknown. This work aimed to describe the global rearrangements of plasma proteome at early pregnancy period and discover potential predictive protein biomarkers of GDM. The results revealed that GDM induced expression level changes of 24 proteins at 16‐18 weeks of gestation. These proteins included pregnancy‐related proteins, protein metabolic process‐related proteins, immunoglobulin, C‐reactive protein, and other proteins. Global correlation analysis indicated existence of some co‐regulated proteins in GDM. Functional annotation analysis indicated that inflammation system, oxidative stress, insulin resistance, blood coagulation, and lipid homeostasis were involved in the GDM induced co‐regulation. This study explored potential predictive biomarkers for GDM and provided a global overview of protein rearrangements induced by GDM.

The 24 changed proteins could be used to explore potential predictive biomarkers for GDM in further studies. The specificity and sensitivity of these discriminating proteins in GDM and control samples indicated their potential values in predictive biomarker study. Compared with our results, some differently expressed proteins in previous studies should be addressed, such as C‐reactive protein, fibrinogen alpha chain, sex hormone‐binding globulin.^10^ Label‐free method used to analyze samples separately in this study could avoid errors induced by peptide tandem mass tag (TMT) labeling and elimination of individual differences by mixing samples. Among the 24 significantly changed proteins, only nine of them were annotated in biological process terms in DAVID 6.8 at 20190527, and the terms included acute‐phase response, female pregnancy, cellular protein metabolic process, cellular response to hormone stimulus, response to glucocorticoid, innate immune response, and response to estradiol. The number of proteins in these terms was limited, and the maximum number was three (detailed data for the GOBP results of the 24 proteins are listed in Table S6). The scattered distribution of these proteins in Gene Ontology biological processes may indicate the mildness of proteomic rearrangements induced by GDM at early second trimester (16‐18 gestation weeks).

Low‐grade inflammation, systemic oxidative stress, and insulin resistance are the characteristics for pregnancies complicated with GDM. Although obesity is associated with low‐grade inflammation, and gestational diabetic women generally have higher body mass indices, chronic subclinical inflammation in women diagnosed with GDM is observed regardless of BMI.^22^ Strong correlations among the inflammation‐related proteins and proteins of blood coagulation, lipid homeostasis membrane, and antioxidative enzymes in this study also indicated proteins have critical role in the development of GDM.

Oxidative stress has been shown to interact with inflammation to regulate disease outcomes.^23^ The changed oxidative stress metabolite biomarkers such as 8‐isopeostane^24^ and malondialdehyde,^25^ plasma oxidation/redox status‐related proteins such as protein carbonyl and nitrotyrosine,^26^ antioxidant activity enzymes such as paraoxonase1 (PON1)^26^ and glutathione peroxidase (GPX),^25^ glycation end products (AGEs) such as N‐ε‐carboxy‐methyl‐lysine (CML).^27^ In our study, global proteomic revealed the involvement of antioxidation proteins in co‐regulations, and positive correlations with blood coagulation, inflammation system, and lipid homeostasis in GDM.

Insulin resistance is mainly caused by imbalance between increased needs of insulin and β‐cell defect in pregnancy complicated with GDM. Additionally, chronic low‐grade inflammation in adipose tissue impairs insulin signaling, which further stimulates expression of genes encoding proteins involved in insulin resistance. In this study, significant change of C‐reactive protein in 16‐18 gestational weeks may verify its role as early pregnancy predictor for developing GDM^28^, ^29^ and co‐regulations of this protein with proteins in inflammation and membrane proteins may indicate the way it works.

Blood coagulation and lipid homeostasis were also documented to be involved in pregnancy complicated with GDM. Hypercoagulability was observed to be enhanced in GDM compared with normal pregnancy group. In precious study, GDM is observed to associate with shortened activated partial thromboplastin time (APTT), increased activity of antithrombin III (ATIII), and a higher plasminogen activator inhibitor (PAI‐1) content levels.^30^ This study may reveal that plasma hypercoagulability in GDM pregnant women was induced by co‐regulations of many blood coagulation proteins. Lipid homeostasis is involved in pregnancy complicated with GDM and whether lipids and lipoproteins could be used as biomarker to predict GDM need further study for the contradictory results. An early study indicated that concentrations of very‐low‐density, low‐density and high‐density lipoproteins, plasma cholesterol, and triglyceride changed along gestation time during pregnancy.^31^ The study on Japanese women at 20‐28 weeks of gestation indicated that the increase of triglycerides, and some apolipoproteins in pregnant women complicated with GDM were not significant.^32^ A research on Chinese women at 24‐28 gestational weeks showed significant increase in total serum cholesterol and did not show significant increase in high‐density lipoprotein cholesterol, low‐density lipoprotein cholesterol, apolipoprotein A‐I, and apolipoprotein B.^33^ In our study, changes of lipid hemostasis‐related proteins were also mild and none of these proteins showed significant change according to fold change and t test. The obvious co‐regulation area of these proteins indicated the sensitivity of global correlation analysis.

The present study lies in the use of a sensitive and reliable LC‐MS/MS technique followed by ELISA confirmatory experiments to analyze plasma samples obtained from GDM cases. Several of the proteins identified in our study have been previously reported by other groups at early GDM biomarkers screening including sex hormone‐binding globulin (SHBG), C‐reactive protein (CRP), serum amyloid P‐component, and fibrinogen alpha chain reported as candidate biomarkers.^28^, ^34^, ^35^ Recently, Mavreli et al^18^ using proteomic analyses identified overexpression of prenylcysteine oxidase 1 (PCYOX1), beta‐ala‐his dipeptidase (CNDP1), extracellular matrix protein 1 (ECM1), basement membrane‐specific heparan sulfate proteoglycan core protein (HSPG2), and thrombospondin 4 (TSP‐4) in the 1st‐trimester maternal plasma of women who were subsequently diagnosed with GDM followed by confirmatory ELISA.

C‐reactive protein (CRP) is routinely used in the diagnosis for monitoring infections especially in the obstetric field.^36^, ^37^ CRP is also an important biomarker for predicting long‐term outcome in inflammatory disease.^38^ Carbone et al^29^ showed that maternal serum levels of CRP during the second trimester of pregnancy represent a useful predictor of maternal adverse outcome occurrence. Fatema et al^28^ showed their data indicate that hsCRP and C‐peptide both are sensitive markers in predicting GDM. Compared with control, our study showed that higher expression of CRP in GDM group determined by LC‐MS/MS and confirmed by ELISA. So, this result indicated that CRP may be acted as one early predictor for GDM early screening.

The insulin‐like growth factor (IGF) axis is an important regulator of fetal growth and development. Abnormal expression of IGF‐I or IGF‐II is associated with severe intrauterine restriction. IGF‐binding proteins (IGFBPs) are inhibitors of IGF actions on metabolism and growth. Currently, there are six kinds of IGFBPs that can complex with IGF‐I and IGF‐II. Qiu et al^39^ showed that lower concentrations of insulin‐like growth factor–binding protein 1 (IGFBP1) in maternal plasma during the second trimester before diagnosis of GDM. Lindsay et al^40^ displayed an inverse relationship with cord blood IGFBP1 of mothers with GDM at term and birthweight. In one recent investigation, IGFBP‐3 as the major binding protein of IGF‐1 in human blood was positively associated with T2DM risk.^41^ Qiu et al^42^ showed increased IGFBP‐4 in the maternal circulation in early pregnancy is associated with the development of fetal growth restriction. Lappas presented that there is a downregulation of maternal plasma levels of IGFBP‐1, IGFBP‐6, and IGFBP‐rP1, and cord plasma levels of IGFBP‐1‐3 and IGFBP‐rP1 in normoglycemic obese pregnancies when compared with normoglycemic non‐obese pregnancies.^43^ IGFBPs have an important role in insulin signaling, enhancing peripheral glucose uptake, decreasing hepatic glucose output and modifying lipid metabolism. Heald et al^44^ demonstrated that IGFBPs correlate with glucose tolerance and insulin resistance and with cardiovascular disease in cross‐sectional study. Heald et al^45^ have shown low circulating IGFBP‐2 is associated with reduced insulin sensitivity. Our study showed that lower expression of IGFBP‐2 in GDM group compared with control by LC‐MS/MS, which also identified by ELISA. Therefore, our results presented that IGFBP‐2 could be used as the early predictive biomarker for GDM prenatal screening.

However, the limited number of cases in present study may result in an overestimation. Further research is required by using a larger population representative of GDM to validate the data obtained in early predicting GDM. Abnormal expression of CRP and IGRBP2 in the first‐trimester maternal plasma of GDM women was identified by ELISA. These proteins displayed great potential to be acted as early predictors for GDM prenatal screening.

## 5 CONCLUSIONS

In summary, our study explored 24 potential predictive biomarkers for GDM and provided a global overview of protein rearrangements induced by GDM at early second trimester. With global correlation analysis, the co‐regulations of inflammation, oxidative stress, insulin resistance, blood coagulation, and lipid homeostasis were also revealed in GDM development. The abnormal expression of CRP and IGFBP2 was verified by ELISA in the first‐trimester maternal plasma in GDM women.

## Supporting information

Table S1

Table S2

Table S3

Table S4

Table S5

Table S6
