# Supplementary material for: Proteome profiling of gestational diabetes mellitus at 16‐18 weeks revealed by LC‐MS/MS
Source: J Clin Lab Anal. 2020 Jun 15;34(9):e23424. doi: 10.1002/jcla.23424 (PMC7521232; doi:10.1002/jcla.23424)
Supplement: Supplementary file 5 — Table S5 [file JCLA-34-e23424-s005.doc]

| **Physiological processes** | **ID** |
| --- | --- |
| inflammation system | O00187 |
|  | O75179 |
|  | O75594 |
|  | O75636 |
|  | P00734 |
|  | P00738 |
|  | P00751 |
|  | P01009 |
|  | P01024 |
|  | P01608 |
|  | P01611 |
|  | P01743 |
|  | P01762 |
|  | P01769 |
|  | P01778 |
|  | P01824 |
|  | P01834 |
|  | P02671 |
|  | P02747 |
|  | P02751 |
|  | P02763 |
|  | P02775 |
|  | P03952 |
|  | P04003 |
|  | P05156 |
|  | P06311 |
|  | P06681 |
|  | P07358 |
|  | P07360 |
|  | P08571 |
|  | P08603 |
|  | P09871 |
|  | P0C0L4 |
|  | P0C0L5 |
|  | P0DJI8 |
|  | P10909 |
|  | P11226 |
|  | P11226 |
|  | P13671 |
|  | P18428 |
|  | P19652 |
|  | P27918 |
|  | P35542 |
|  | P48740 |
|  | P49913 |
|  | P61769 |
|  | P80419 |
|  | Q14624 |
|  | Q99969 |
|  | Q9NPH3 |
|  | Q9NZP8 |
|  | P04431 |
|  | P01764 |
|  | P01716 |
|  | P01612 |
|  | P80748 |
|  | P01596 |
|  | P04211 |
|  | P06889 |
|  | P19320 |
|  | P43121 |
|  | P01781 |
|  | P01604 |
|  | P01714 |
|  | P01602 |
|  | P01610 |
|  | P01601 |
|  | P01613 |
|  | P23083 |
|  | P06310 |
|  | P01708 |
|  | P10643 |
|  | P04433 |
|  | P01825 |
|  | P01620 |
|  | O43866 |
|  | P01621 |
|  | P01779 |
|  | P01624 |
|  | P06309 |
|  | P01625 |
|  | P01765 |
|  | P01766 |
|  | P01775 |
|  | Q96PD5 |
|  | P01772 |
|  | P01763 |
| membrane | Q8N0X4 |
|  | Q86YV9 |
|  | O14786 |
|  | Q8TF62 |
|  | Q16706 |
|  | Q9P2P1 |
|  | P01871 |
|  | P13473 |
|  | O00533 |
|  | P14151 |
|  | P04220 |
|  | O00391 |
|  | Q13219 |
|  | P09172 |
|  | Q9C0B1 |
|  | P55290 |
|  | Q14126 |
|  | Q9NPY3 |
|  | P54108 |
|  | P16070 |
|  | Q16853 |
|  | Q9UIQ6 |
|  | P07359 |
| lipid homeostasis system | P02647 |
|  | O14791 |
|  | P02652 |
|  | P02655 |
|  | P02656 |
|  | P04114 |
|  | P06727 |
|  | P08519 |
|  | P55056 |
|  | P55058 |
|  | Q13790 |
|  | Q6Q788 |
| blood coagulation | P01042 |
|  | P05546 |
|  | P04070 |
|  | P12259 |
|  | P00742 |
|  | P00740 |
|  | Q76LX8 |
|  | P40197 |
|  | P03951 |
|  | P05160 |
|  | P00748 |
|  | P00747 |
|  | P08709 |
|  | Q96IY4 |
|  | P07225 |
| acetylation proteins | P07737 |
|  | P62937 |
|  | P18206 |
|  | Q9Y490 |
|  | P04075 |
|  | P68032 |
|  | P21333 |
|  | P67936 |
|  | P37802 |
|  | P23528 |
|  | Q15942 |
|  | Q00341 |
