# Supplementary material for: Proteome profiling of gestational diabetes mellitus at 16‐18 weeks revealed by LC‐MS/MS
Source: J Clin Lab Anal. 2020 Jun 15;34(9):e23424. doi: 10.1002/jcla.23424 (PMC7521232; doi:10.1002/jcla.23424)
Supplement: Supplementary file 6 — Table S6 [file JCLA-34-e23424-s006.docx]

| Term | Count | % | PValue | Genes | List Total | Pop Hits | Pop Total | Fold Enrichment | Bonferroni | Benjamini | FDR |
| --- | --- | --- | --- | --- | --- | --- | --- | --- | --- | --- | --- |
| GO:0006953~acute-phase response | 3 | 13.04347826 | 7.86E-04 | P0DJI9, P02743, P02741 | 19 | 39 | 16792 | 67.98380567 | 0.133944257 | 0.133944257 | 0.965914993 |
| GO:0007565~female pregnancy | 3 | 13.04347826 | 0.004021705 | P20742, P11465, P18065 | 19 | 89 | 16792 | 29.79065642 | 0.521670738 | 0.308386479 | 4.855572346 |
| GO:0044267~cellular protein metabolic process | 3 | 13.04347826 | 0.006960343 | P02671, P18065, P02743 | 19 | 118 | 16792 | 22.46922391 | 0.721460762 | 0.3469264 | 8.265450748 |
| GO:0032870~cellular response to hormone stimulus | 2 | 8.695652174 | 0.047177366 | P10912, P18065 | 19 | 45 | 16792 | 39.27953216 | 0.999855719 | 0.890402163 | 44.94864825 |
| GO:0051384~response to glucocorticoid | 2 | 8.695652174 | 0.067463206 | P10912, P18065 | 19 | 65 | 16792 | 27.19352227 | 0.999997189 | 0.922415541 | 57.79829692 |
| GO:0045087~innate immune response | 3 | 13.04347826 | 0.076431762 | P02671, P01871, P02743 | 19 | 430 | 16792 | 6.165973072 | 0.99999952 | 0.911528344 | 62.54662343 |
| GO:0032355~response to estradiol | 2 | 8.695652174 | 0.09322543 | P10912, P18065 | 19 | 91 | 16792 | 19.42394448 | 0.999999983 | 0.922569671 | 70.14246582 |
